# Bridging the B Cell Gap: Novel Technologies to Study Antigen-Specific Human B Cell Responses

**DOI:** 10.3390/vaccines9070711

**Published:** 2021-07-01

**Authors:** Henry A. Utset, Jenna J. Guthmiller, Patrick C. Wilson

**Affiliations:** 1 Department of Medicine, Section of Rheumatology, University of Chicago, Chicago, IL 60637, USA; hutset@medicine.bsd.uchicago.edu (H.A.U.); wilsonp@uchicago.edu (P.C.W.); 2 Committee on Immunology, University of Chicago, Chicago, IL 60637, USA

**Keywords:** human B cells, B cell repertoires, humoral immunity, broadly neutralizing antibodies, RNA-sequencing, humanized mice, germinal center, bone marrow, lymph node, fine needle aspirates

## Abstract

The generation of high affinity antibodies is a crucial aspect of immunity induced by vaccination or infection. Investigation into the B cells that produce these antibodies grants key insights into the effectiveness of novel immunogens to induce a lasting protective response against endemic or pandemic pathogens, such as influenza viruses, human immunodeficiency virus, or severe acute respiratory syndrome coronavirus-2. However, humoral immunity has largely been studied at the serological level, limiting our knowledge on the specificity and function of B cells recruited to respond to pathogens. In this review, we cover a number of recent innovations in the field that have increased our ability to connect B cell function to the B cell repertoire and antigen specificity. Moreover, we will highlight recent advances in the development of both ex vivo and in vivo models to study human B cell responses. Together, the technologies highlighted in this review can be used to help design and validate new vaccine designs and platforms.

## 1. Introduction

The humoral arm of the immune system plays a key role in vaccine-mediated immunity through the production of protective high-affinity neutralizing and non-neutralizing antibodies against diverse pathogens [1]. The antigen reactivity and binding affinity of serum antibodies generated in response to vaccination or infection has been studied for decades using techniques such as enzyme-linked immunosorbent assays (ELISAs) or surface plasmon resonance (SPR), in addition to neutralization experiments to test the protectiveness of polyclonal sera against pathogens. These techniques, however, fail to provide information about the B cell clones involved in the immune response or the phenotypes of B cells recruited into the response. These questions have, in turn, been probed using monoclonal antibodies (mAbs) generated from single B cells [2] isolated following antigen exposure [2,3,4], allowing for inquiry into the activity of individual antibodies from both peripheral and tissue-resident B cells. This cloning technology has also allowed for repertoire analysis to investigate the makeup of the polyclonal response to antigen exposure. B cells have also been isolated out of biopsied tissues or lymph nodes. However, biopsied tissues can be abnormal and thus may not perfectly represent the physiological human immune system. Additionally, to model the human immune system in vivo, mice have been generated with severe congenital immunodeficiencies that allow for the transplantation of human bone marrow [5]. However, these models lack secondary lymphoid structures, limiting their utility in probing human B cell responses to antigen.

Recent developments in B cell technologies have allowed for investigation into novel dimensions of B cell biology. Innovative new techniques have allowed for the connection of the B cell repertoire to serum antibody responses [6,7], or the connection of B cell receptor (BCR) specificity to the function of heterogenous B cell populations [8]. Additionally, improved in vivo and ex vivo approaches have created new opportunities for the longitudinal study of the human immune system under truer physiological conditions, including the development of ex vivo organoids, bone marrow mimics and refined humanized mouse models [9,10,11]. The development of fine-needle aspiration techniques of human lymph nodes and bone marrow has also opened new approaches to the study of the in vivo behavior of human B cells in humans following vaccination or infection [12,13,14]. In this review, we will discuss the emerging technologies and techniques to investigate B cell specificity and function and how these new approaches can provide critical insight into B cell biology in the context of infection, vaccination, and autoimmunity.

## 2. Connecting Antigen Specificity to B Cell Heterogeneity

Although much work has been done to investigate the antigen specificities of B cells and B cell differentiation, little is known about the relationship between the two or how they may differ in the context of heterogenous B cell populations in distinct tissues. The relationship between antigen specificity and B cell identity can have significant clinical ramifications, such as the discovery of antigen-specific atypical memory B cells (AtM) in certain cases of chronic pathogen exposure such as the malaria-causing parasite *Plasmodium falciparum* [15] or HIV [16] that exhibit an exhaustion phenotype and differ in their immunoglobulin (Ig)gene usage from classical memory B cells [15]. The role played by AtMs is unclear, and further complicated by their presence in individuals without chronic antigen exposure [17]. There is also increasing evidence of B cell heterogeneity across tissues [18,19,20], but little work has been done to investigate any connections between that heterogeneity and the specificities of those B cells. Notably, only relatively low-throughput methods have been available for the sequencing of paired antibody heavy/light chains combined with the antigen specificity of that antibody, typically by screening of mAbs [21]. The lack of a high-throughput technology has made exploring connections between antigen specificity and B cell subset identity difficult.

**Figure 1:**
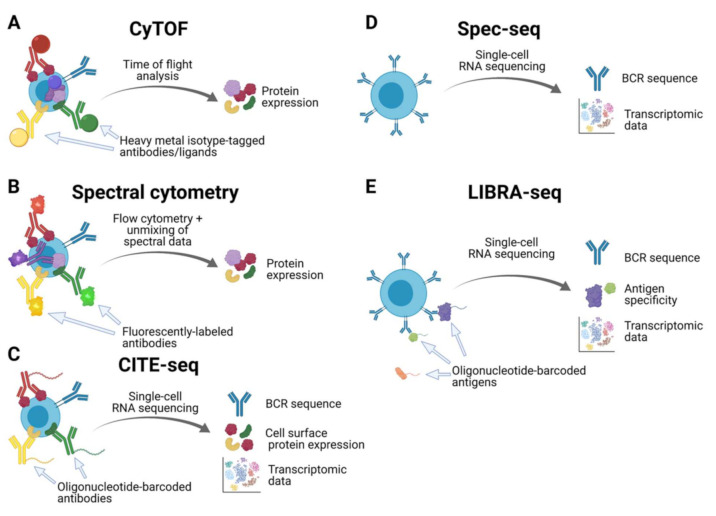
Inputs and outputs of various techniques for single-cell phenotyping of B cells. (**A**) Cytometry by time of flight (CyTOF) and (**B**) spectral flow cytometry are high-throughput mechanisms to determine cellular phenotypes by protein expression levels. (**C**) Cellular indexing of transcriptomics and epitopes by sequencing (CITE-seq) can reveal protein expression via single-cell RNA-seq, which also reveals the transcriptome and BCR sequences. (**D**) Specificity sequencing (spec-seq) can assess both transcriptomic and BCR sequences, whereas (**E**) linking B cell receptor to antigen specificity through sequencing (LIBRA-seq) can connect antigen specificity to single-cell RNA sequencing data. Both spec-seq and LIBRA-seq can be used in conjunction with CITE-seq protocols.

The advent of cytometry by time of flight (CyTOF, Figure 1A) has been a valuable advance in the field of cytometry and single-cell feature analysis to investigate B cell heterogeneity. Similar to traditional flow cytometry, CyTOF allows for the characterization of cell surface and intracellular proteins. However, rather than labeling antibodies with fluorescent tags, antibodies are instead conjugated with metal isotypes [22]. This approach avoids the problematic signal overlap that occurs when using many fluorescently labeled antibodies in traditional flow cytometry. Because of the abundance of different metallic labels available for use, it is possible to use more than 40 metal-tagged antibodies, nucleic acid intercalators or other ligands of interest in a single run [22]. This presents a significant advance in the vaccine field’s ability to phenotypically characterize different B cell subsets, such as the mapping of B cell subsets across tissues by protein expression [23]. Of note, CyTOF markers are not limited to the cell surface but are also able to interact with targets inside the cell or even within organelles, further broadening the possibilities for cellular inquiry [24]. However, with recent advances in optical technology, it is possible that spectral flow cytometry (Figure 1B) will become the most popular technology in the field of cytometry and single-cell feature analysis. Spectral flow cytometry allows for similarly multiplexed investigation of cell phenotypes as CyTOF without the need for unusual metal isotype reagents, making use of more familiar traditional flow cytometry reagents instead [25,26]. Notably, recently developed analysis algorithms have shown comparable results between spectral flow cytometry and CyTOF data, further driving the adoption of spectral flow cytometry as a mainstay of single-cell phenotypic characterization [27].

Cellular indexing of transcriptomes and epitopes by sequencing (CITE-seq, Figure 1C) is another recently developed approach to interrogate cell surface features by RNA-seq. CITE-seq uses oligonucleotide-labeled antibodies to characterize the expression of cell surface proteins of interest in a highly multiplexed manner [28]. These oligonucleotides are compatible with most single-cell RNA sequencing techniques, allowing for the detection of oligo-tagged antibodies, and therefore, measurement of cell surface protein expression levels, in parallel with current single-cell RNA-sequencing protocols [28]. CITE-seq is thus a powerful technology that is easy to pair alongside transcriptomic analysis in experiments investigating the characteristics of B cells that are activated in response to infection or vaccination.

Novel approaches to explore the relationship between antigen specificity and B cell identity such as specificity-sequencing (spec-seq, Figure 1D) [29] or microfluidics-based approaches [30] including the use of commercial apparatuses, such as the 10xGenomics platform in combination with next-generation or long-read sequencing techniques such as Pacific Biosciences’ SMRT sequencing or Oxford Nanopore Technologies, are coupled approaches to sequence both the BCR and transcriptome from the same cell. Specifically, the spec-seq pipeline includes single-cell sorting of B cells or plasmablasts from peripheral blood mononuclear cells (PMBCs) followed by single-cell RNA sequencing and immunoglobulin gene cloning and expression [31]. This process provides insight into ongoing cellular processes and B cell subset identity through transcriptomic data, as well as BCR sequence through the many antibody RNA transcripts present such as from plasmablasts induced by vaccination. Using the BCR sequence, it is then possible to express monoclonal antibodies of the same specificity and explore their antigen reactivity [29]. This technology was used recently to compare the transcriptional profiles of plasmablasts of distinct isotypes (IgG or IgA) in response to influenza vaccination and separated by vaccine reactivity, revealing that there is little difference at the transcriptional level between IgG and IgA plasmablasts induced by vaccination [29]. However, spec-seq is still a relatively low-throughput technique, as characterizing the antigen reactivity of recombinant monoclonal antibodies produced from the uncovered BCR sequences is a labor-intensive process. 

By contrast, linking B cell receptor to antigen specificity through sequencing (LIBRA-seq, Figure 1E) allows for the high-throughput analysis of antigen-specific B cell responses to infection and vaccination by simultaneously mapping BCR sequence to antigen specificity and B cell transcriptomic data to allow for B cell subset identification via microfluidics-based approaches [8]. This is accomplished by antigen-specific cell sorting using recombinant protein antigens conjugated to DNA oligonucleotide “barcodes” followed by single-cell RNA sequencing. The transcriptomic data provide information about B cell subset identity as well as BCR sequence, while the antigen barcode(s) present indicate which antigen(s) a given B cell is able to bind [8]. Notably, LIBRA-seq can also reveal broadly reactive BCRs, as B cells may bind to multiple DNA-barcoded recombinant proteins, such as distinct hemagglutinin (HA) proteins from influenza viruses. Therefore, LIBRA-seq allows for the rapid characterization of B cell responses to novel vaccine immunogens or antigens following infection, which can yield the discovery of previously unknown broadly reactive antibodies or tie B cell differentiation to specificity [32]. This powerful technology was also used to uncover antigen-specific memory B cell heterogeneity following severe acute respiratory syndrome coronavirus-2 infection [32]. Moreover, LIBRA-seq has been used to reveal that AtM cells are present in both antigen-naïve individuals as well as those experiencing chronic *Plasmodium falciparum* infections [17]. Together, these different single-cell technologies are capable of illuminating the specificity and function of human B cells (Figure 1). However, each has its strengths and limitations, so selecting the appropriate technique to answer the specific question of interest is important. The strengths and limitations of each technique with respect to the phenotypes revealed in each case are summarized in Table 1. Of note, the RNA-seq-based approaches all allow for the cloning of monoclonal antibodies and provide insight into gene expression levels, but reagents for these experiments can be expensive. CyTOF and spectral flow cytometry are less expensive and allow for single cell phenotyping, but do not provide BCR sequence for any subsequent characterization of monoclonal antibodies. In contrast, RNA-seq platforms, with the exception of CITE-seq, provide limited insight into phenotypic features of B cells and are costly on a per sample basis.

In addition to the techniques summarized above, newer technologies to reveal the spatial dynamics of cell populations have recently been developed, including spatial transcriptomics of whole tissues as well as multilayer microscopy by co-detection by indexing (CODEX) imaging, that have great potential to reveal migration patterns of maturing B cells within lymphatic tissues. The rapidly evolving field of spatial transcriptomics can reveal single-cell transcriptomic levels of distinct mRNAs in distinct tissue niches [33,34,35,36]. Moreover, CODEX imaging reveals the cellular phenotypes of distinct niches within whole tissue by repeated imaging of distinct markers [37]. Utilization of spatial transcriptomics and CODEX imaging hold great promise in dissecting the nuances of B cell heterogeneity and B-T cell interactions within germinal centers, which can provide insight on the generation of long-lived humoral immunity. However, both techniques remain both technically and financially constraining, with the need for specific expertise in these techniques. However, the commercialization of spatial transcriptomic techniques by 10x Genomics and Nanostring and CODEX imaging by Akoya Biosciences will undoubtedly make these techniques more accessible to researchers in all biomedical research fields.

## 3. Connecting B Cell Repertoires to Serum Antibody Specificities

Electron microscopy polyclonal epitope mapping (EMPEM) is a technique that may be used to characterize the epitope targeting of polyclonal sera in response to vaccination or infection. Since its development and initial application to characterizing polyclonal antibody (pAb) responses to HIV-1 BG505 Envelope trimer immunizations [7], it has been used to investigate the epitope targeting of human pAb responses to H5N1 vaccination [6] as well as for other studies of HIV vaccine development [38,39,40]. PAb responses had previously been probed using techniques such as ELISA, but this does not provide information about epitope targeting. Using EMPEM, it is possible to uncover epitope targeting in polyclonal sera, although this information is decoupled from the sequences of the individual mAbs observed participating in the pAb response. With the advent of high-throughput immunoglobulin sequencing (Ig-seq), however, it is possible to sequence the BCRs of many B cells activated in response to infection and vaccination [41]. In cases where there is a known association between a particular V gene and an epitope, such as VH1-69 and the stalk epitope of influenza hemagglutinin, it may be possible to make inferences between Ig-seq data and EMPEM structures. Additionally, for epitopes that require affinity maturation, longitudinal studies combining EMPEM and Ig-seq may provide insight into the timepoint at which polyclonal sera begin to measurably target that epitope, such as the delay observed in the targeting of the influenza H5 head domain in response to a chimeric H5N1 vaccine in H5-naïve individuals [6]. The corresponding expansion of B cell clones revealed by Ig-seq in concert with EMPEM pAb targeting at that timepoint may lend insight into the clones responsible for targeting that epitope.

## 4. Animal Models to Study the Humoral Immune System

Human immune system (HIS) mice may be used to explore the behavior of human immune cells and immune tissues in vivo. HIS mice are generated by xenografting human hematopoietic stem cells and other human tissues into mice with severe genetic immunodeficiencies, inducing tolerance to the xenografted tissues while simultaneously eliminating murine immune cell contributions to experimental observations. Various immunodeficient mice exist for such experiments and typically use mutations in the interleukin-2 (IL-2) receptor γ-chain locus and in the receptor rearrangement proteins *Rag1*, *Rag2*, and *Prkdc* that lead to limited, functionally impaired B and T cell populations and inhibit natural killer (NK) cell development [5]. However, these mice fail to generate functional secondary lymphoid tissues (SLT) due to defective IL-7 signaling associated with the *Il2rg* mutation [42]. Because SLT are key sites where antigen-specific B and T cells interact and form germinal centers, these models are not useful for probing questions related to affinity maturation, a process of great interest in responses to vaccination or infection. This weakness has recently been resolved by the induction of thymic-stromal-cell-derived lymphopoietin (TSLP) expression in Balb/c *Rag*^–/–^ Il2rg^–/–^ Sirpa^NOD^ (BRGS) mice, referred to as BRGST mice [43]. Because of its functional similarity to IL-7 in SLT but lack of reliance on a functional *Il2rg* gene, the induced TSLP expression rescues SLT development in BRGST mice. As a result, BRGST mice successfully develop physiologically organized SLT, including a larger thymus, as well as mount an improved adaptive immune response to vaccination relative to non-TSLP-expressing mice [43]. This novel model holds great promise for future investigations of human immune cells, particularly B cells and T follicular helper (Tfh) cells, in a more physiologically relevant system. These mice can serve as a valuable preclinical model for determining whether novel immunogens can activate and recruit human naïve B cells against conserved, immunosubdominant epitopes, including those found on the influenza virus hemagglutinin stalk domain and the CD4 binding site of HIV gp120.

BCR knock-in (BCR-KI) mice are an established experimental model, of interest both for understanding antigen-specific B cell development and function in vivo and to test immunogens that can engage particular BCRs. Immunogens able to engage the germline precursors of known broadly neutralizing antibodies (bnAbs) are of great interest for vaccine design. Recently, BCR-KI models have been used to validate novel immunogens that are meant to recruit bnAb precursor B cells against HIV-1 [44]. Notably, many bnAbs against HIV require extensive affinity maturation to acquire their neutralization breadth. BCR-KI mice allow for the interrogation of how immunogens can drive the activation of low-affinity germline BCR-KI B cells and promote affinity maturation to become broadly neutralizing. This approach is also valuable for designing universal influenza virus immunogens that are able to recruit known B cells expressing broadly neutralizing human antibodies into the immune response, such as HA stalk-targeting clones that are rarely induced by current seasonal vaccines or natural infection [45]. Moreover, many bnAbs demonstrate features of autoreactivity and polyreactivity [46,47,48]. BCR-KI mice can serve as valuable tools to understand how potentially autoreactive B cell clones develop and are selected into the bnAb pool. 

BCR-KI mice have been traditionally generated via gene modification in embryonic stem (ES) cells. However, these methods require significant technical skill and equipment as well as long periods of time and planning. The CRISPR/Cas9 knock-in approach facilitates the generation of BCR-KI mice because of its speed and simplicity [49,50]. Using ES gene modification approaches, it could take as long as two years to produce homozygous animals [51]. In contrast, the CRISPR/Cas9 approach is able to insert the prearranged BCR, transforming this previously laborious undertaking into a simple, fast process that will save research groups a great deal of time, resources and reagents, allowing for the easy generation of a greater number of KI models than was previously feasible [49,50].

## 5. Ex Vivo Models to Study the Human Humoral Immune System 

An alternative model for the interrogation of the human immune system outside of humanized mice is ex vivo organoids, namely tonsils. By using tonsil organoids as an experimental system, it is possible to effectively study key aspects of the immune response to vaccination in humans, such as affinity maturation, plasmablast differentiation, class-switch recombination, and memory induction within germinal center reactions [9]. As vaccine candidates tested in animals often fail in human clinical trials [52,53], ex vivo culturing of human lymphoid tissues can serve as an additional pre-clinical model to understand how human B and T cells can engage a given vaccine candidate. This model is useful for longitudinal studies, as it is possible to remove cells at multiple timepoints for sequencing or imaging, allowing researchers to track B cell differentiation markers over time as the humoral response matures [9]. Moreover, these tonsil organoids allow for affinity maturation analysis; after depleting the initial population to remove non-naïve B cells, researchers can analyze the clones that are expanded in response to antigen stimulation and identify mutations that arise over the duration of the germinal center reaction to enhance the binding affinity of the antibodies [9]. Moreover, ex vivo organoids allow for the study of Tfh cells within germinal centers and interrogation-linked recognition of antigen by B and Tfh cells. Notably, tonsils are a relatively abundant tissue due to the volume of tonsillectomy surgeries performed in the developed world and are therefore accessible to many laboratories associated with healthcare centers. However, it is also worth noting that in most cases tonsillectomies are performed because of health issues, making this system an imperfect model for a truly physiological human immune system. Alternatively, peripheral lymph nodes can be used, although these tissues are rarely collected from healthy donors. This method is also amenable to experiments that wish to test different drug or adjuvant conditions [9,54].

The bone marrow is another site of great interest in responses to vaccination because it is where long-lived plasma cells (LLPCs) predominantly reside [55,56]. LLPCs are important because of the key role they play in mediating long-lived humoral immunity generated by vaccination or infection [57,58,59]. While some antigens induce stable antibody titers, produced by LLPCs for decades, others induce a less durable response [60]. As such, investigation into the determinants associated with LLPC fate and maintenance are important for consideration in vaccine design. By the use of ex vivo bone marrow mimics, researchers can understand the development and biology of LLPCs without the need for recurrent bone marrow aspirates. Crucial factors to create an ex vivo bone marrow environment mimic include secreted molecules from primary bone marrow mesenchymal stromal cells (MSC), a proliferation-induced ligand (APRIL), and oxygen-poor conditions [10,11]. Using these conditions, it is possible to conduct experiments on LLPCs or plasmablasts that mimics the physiological bone marrow environment, opening new avenues of inquiry into the nature of LLPC behavior, survival, and fate decisions.

## 6. Tracking B Cell Function In Vivo

Most investigation into B cell biology in the modern era has been done by isolating B cells from the blood using single cell sorting techniques when probing questions about somatic hypermutation or the antigen specificities of monoclonal antibodies. These types of experiments have granted and continue to grant many novel discoveries, but do not provide insight into the in vivo behavior of B cells, including the entry and participation of B cells into germinal centers. These sites represent key locations for affinity maturation to generate a highly protective antibody response and are thus of great interest for vaccine development. Germinal centers may form in ex vivo models of the human immune system, such as the previously mentioned tonsil organoids. However, most vaccines do not stimulate cells in the tonsils, but rather cells in the draining lymph nodes from the site of vaccination [61]. As such, tonsil model systems are not necessarily representative of physiological vaccine responses. Additionally, tonsils serve as the first site of immune activation against inhaled or ingested pathogens, making them a site of chronic stimulation due to the constant antigen exposure. This constant stimulation can make tracking the maturation of an antigen-specific B cell response more difficult. By contrast, techniques that can track B cell responses within lymph nodes avoid issues associated with chronic antigen exposure. The axillary lymph nodes are the natural site where cells are stimulated by intramuscular vaccinations, making them a more relevant model for responses to novel vaccine designs. Lastly, as the lymph nodes are not removed, it possible to observe how the immune response to vaccination develops over time.

Using ultrasound-guided fine-needle aspirates of the draining lymph nodes, it is now possible to safely and minimally invasively probe germinal centers in vivo during the course of the immune response to vaccination in humans [14,62]. Fine-needle aspiration of the draining lymph nodes has recently been used to show that both broadly cross-reactive memory B cells as well as strain-specific naïve-like B cells participate in human germinal center responses following influenza virus vaccination [12]. Because of the large number of mutations required for bnAbs against HIV to acquire their activity, monitoring immunogens’ ability to generate and sustain germinal center responses over time is of interest for vaccine design. In one recent report, fine needle aspirates were used to follow the maturation of neutralizing antibody responses to HIV immunogens using a slow administration technique, finding that slower administration facilitates the generation of neutralizing antibodies in non-human primates [38]. Tracking germinal center responses is of great interest in HIV vaccine development, with multiple other studies making use of fine needle aspirates since its development to monitor lymph nodes for the recruitment and affinity maturation of B cell clones of interest [63,64]. Ultrasound-guided fine-needle aspirates therefore represent a significant advance in the vaccine field’s ability to investigate human rather than murine germinal center responses to antigen exposure and track B cells in vivo over time. Fine needle aspirates also allow for interrogation of B cell and T cell interactions in response to vaccination, including insight into Tfh cell populations recruited into germinal centers during the immune response. In conjunction with studying B cell responses, lymph node aspirates allow for simultaneous interrogation of Tfh clonal dynamics over time, transcriptional and phenotypic features, antigen-specificity, and potentially linked recognition of antigen by B and T cells. Additionally, as with fine needle aspirates of the lymph nodes, it is similarly possible to take fine needle aspirate samples of the bone marrow, allowing for the characterization of in vivo human B cell responses over time in the bone marrow compartment. Using bone marrow fine needle aspirates, new investigation into the binding affinities, epitope specificities, and maintenance of LLPCs over time is possible, such as one recent study that found that LLPCs induced by influenza vaccination decline within a year after vaccination [13]. This finding suggests that the bone marrow environment alone is insufficient to maintain the survival of plasma cells that immigrate to the bone marrow; the discovery of additional factors driving the survival of new bone marrow immigrants will be a crucial issue of interest for studies in vaccine development.

Lastly, techniques to investigate the spatial organization of single-cells within tissue have continued to yield increasingly high-resolution insight into the transcriptomic profiles of microanatomical structures [33,65]. This presents exciting opportunities to study the localization of memory B cells or LLPCs following vaccination or infection to identify survival niches. For example, as mentioned above, it has been shown that LLPCs induced by influenza vaccination decline within one year [13]. Revisiting this phenomenon using spatial transcriptomics may reveal key combinations of spatial localization factors, gene expression, and/or BCR specificities that determine the survival or loss of the induced LLPCs.

## 7. Conclusions

**Figure 2:**
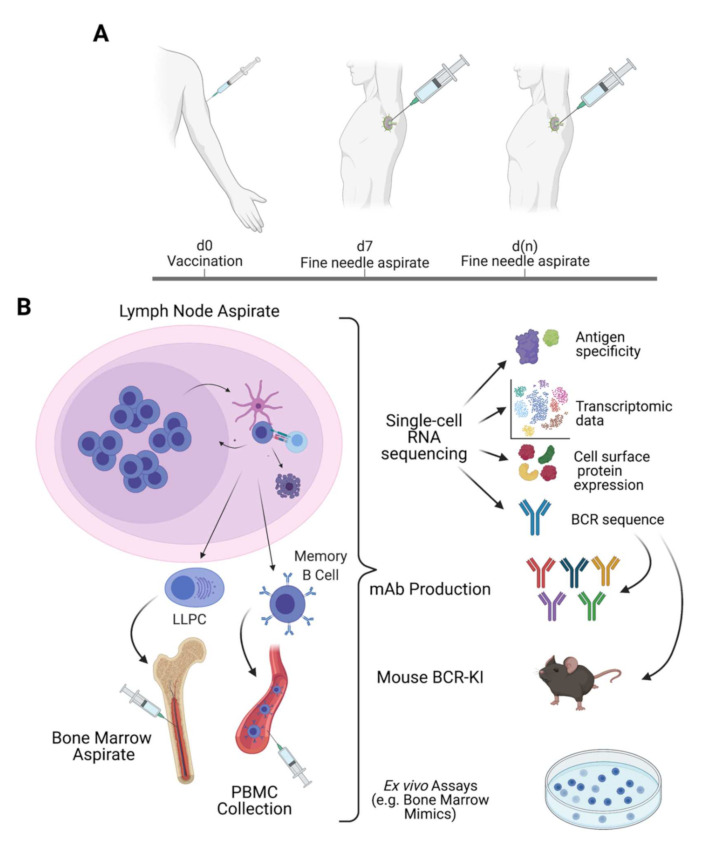
Example of generic potential study making use of longitudinal fine needle aspirates of the lymph nodes (**A**) and the bone marrow to investigate B cells in different compartments over time in combination with single-cell RNA-sequencing methods, which can be used to make mAbs and BCR-KI mice, as an ex vivo bone marrow mimic experiment (**B**).

In summary, the novel techniques and innovations discussed in this review have great potential to deepen our understanding of B cell biology at an unprecedented pace. With relative ease, one could sew together a number of these techniques into a striking, multifaceted series of experiments (Figure 2) to comprehensively analyze the B cell response to vaccination. Taking fine needle aspirates at multiple timepoints, one can monitor for germinal center induction and affinity maturation in the lymph nodes or for new bone marrow immigrants and their continued survival over time [12,13,14]. Using LIBRA-seq, one can uncover the antigen specificity, clonal identity, and transcriptomic profiles of cells isolated from fine needle aspirate samples or from traditional peripheral blood samples [8]. With the novel CRISPR/Cas9 approach to BCR knock-in mice, broadly neutralizing clones identified through LIBRA-seq can then be rapidly made into BCR-KI mouse models to test for their ability to be recruited by extant or novel vaccine candidates [44,49,50]. If successful, these immunogens could then also be tested for their ability to recruit and affinity mature those broadly neutralizing human B cell clones in a competitive polyclonal context using BRGST mice transplanted with human HSCs [43]. Alternatively, those cells could also be tested for their ability to survive in an ex vivo bone marrow mimic culture [10,11] or for their ability to enter germinal centers in an ex vivo tonsil organoid model [9]. It is an exciting time to be in the field of vaccinology, and the technologies available today to design and validate novel immunogens for vaccines against established as well as emerging pathogens hold more promise than ever to eliminate these significant threats to public health.

## Figures and Tables

**Table 1:** Comparison of the advantages and limitations of single-cell phenotyping of B cells using CyTOF, spectral cytometry, CITE-seq, Spec-seq and LIBRA-seq.

	CyTOF	Spectral Cytometry	CITE-seq	Spec-seq	LIBRA-seq
BCR sequence	No	No	Yes *	Yes	Yes
Antigen specificity	No	No	Yes *	No	Yes
Transcriptomic data	No	No	Yes *	Yes	Yes
Cell surface protein expression	Yes	Yes	Yes	No	No
Intracellular protein expression	Yes	Yes	No	No	No

* In combination with spec-seq and LIBRA-seq methods.

## Data Availability

Not applicable.
